# α-Amylase inhibitory activity of some traditionally used medicinal species of Labiatae

**DOI:** 10.1186/s40200-014-0114-1

**Published:** 2014-12-02

**Authors:** Hedieh Safamansouri, Marjan Nikan, Gholamreza Amin, Parisa Sarkhail, Ahmad Reza Gohari, Mahdieh Kurepaz-Mahmoodabadi, Soodabeh Saeidnia

**Affiliations:** Medicinal Plants Research Center, Tehran University of Medical Sciences, P. O. Box 14155–6451, Tehran, Iran; Department of Pharmacognosy, Faculty of Pharmacy, Tehran University of Medical Sciences, Tehran, Iran; Pharmaceutical Sciences Research Center, Tehran University of Medical Sciences, Tehran, Iran; Division of Pharmacy, College of Pharmacy and Nutrition, University of Saskatchewan, Saskatoon, Canada

**Keywords:** α-amylase inhibitory, *Phlomis*, *Satureja*, *Salvia*, *Scutellarua*, *Stachys*, *Hymenocrater*

## Abstract

**Background:**

Natural α-amylase inhibitors of herbal origin are an attractive therapeutic approach to control post-prandial hyperglycemia via reducing the glucose release from starch and delaying carbohydrate absorption. These compounds are able to inhibit the activity of the carbohydrate hydrolyzing enzymes in the small intestine and potentially useful in control of diabetes. The enlarged Lamiaceae (Labiatae) family contains about 6,900 to 7,200 species worldwide and many species of this family possess medicinal properties and have been used traditionally for treatment of chronic illnesses including diabetes.

**Methods:**

In the present study particular species of Labiatae family from the genera, *Phlomis, Satureja, Salvia, Scutellarua, Stachys* and *Hymenocrater,* which are growing wildly in Iran, selected to evaluate for possible in vitro α-amylase inhibitory activity, compared to acarbose as a positive control.

**Results:**

The inhibitory activities of all the herbal extracts were varied from 1.9 to 18.6 (IC50, μg/mL). Additionally, the ethyl acetate extract of *P. bruguieri* (IC50 = 1.9 μg/mL) and the butanol extract of *P. persica* (IC50 = 3.6 μg/mL) exhibited the lowest IC50 values among all the species as the most potent herbal extracts, while the inhibitory activity of *S. sahendica* and *S. macrosiphon* (ethyl acetate extracts) as well as *P. caucasica* (butanol extract) on α-amylase enzyme was observed as weak and did not reach at least to the 50% of the enzyme inhibition level.

**Conclusions:**

Taking together, *P. bruguieri* and *P. persica* among the *Phlomis* species can be the promising sources of α-amylase inhibitors. However, *P. rigida, S. bizantina* and *H. bituminosus* that exhibited moderate activity can be stand on second level of interest.

## Background

Diabetes syndrome is a metabolic disorder, which causes congenital (type I insulin-dependent) or acquired (type II noninsulin-dependent) diabetes mellitus. In this syndrome, the metabolism of food changes and the blood glucose concentration increases [1]. More than 346 million people worldwide are suffering from diabetes. According to the figures reported by the World Health Organization (WHO), 3.4 million people died from consequences of high blood sugar in 2004. As a matter of fact, diabetes in the world, especially in developing countries, is growing. There are many reasons for that but the most important one is obesity and lack of exercise [2]. More than 80% of diabetes deaths occur in low and middle income countries [3]. Diabetes is predicted to become the seventh leading cause of the death in the world by the year 2030 [4]. Healthy diet, regular exercise, maintaining proper body weight and avoiding tobacco use can prevent or delay type II diabetes [5]. Various therapies such as diet, exercise and drugs are now recommended for diabetes. Mechanism of medication for lowering the blood sugar is different. Drugs are not effective in the treatment of diabetes they just lower the blood glucose [6].

For this reason, a majority of both physicians and patients tend to apply complementary and alternative therapies such as herbal medicines to control blood sugar. One of the important ways to treat type 2 diabetes is lowering blood glucose levels after a meal. This means that after eating hyperglycemia is reduced by inhibition of carbohydrate hydrolyzing enzymes such as α-amylase and the absorption of glucose slows down from the gastrointestinal tract [7]. It has been reported that the extracts of medicinal plants that showed inhibitory properties of α-amylase enzymes were able to reduce the absorption of glucose, where they were administered with meal [8]. In the recent years, medicinal plants with lowering blood glucose activity have been attractive for the scientists in this area and studied in diabetes treatment [9].

In the present study, we aimed to evaluate the activity of some herbal extracts for their probable α-amylase inhibitory activities in order to find the promising influence inhibitors to control after meal- hyperglycemia as a way to treat and control type II diabetes with fewer side effects. The medicinal plants used in this study are indicated in Table 1. All the plant species belong to Lamiaceae (Labiatae) family.

**Table 1:** **Plants names and herbarium voucher numbers**

**Sample numbers**	**Plant species**	Herbarium voucher numbers*
**1**	*Phlomis bruguieri*	1581
**2**	*Phlomis kurdica*	1582
**3**	*Phlomis rigida*	1557
**4**	*Phlomis olivieri*	1631
**5**	*Phlomis caucasica*	1648
**6**	*Phlomis anisodonta*	1634
**7**	*Phlomis persica*	1610
**8**	*Satureja sahendica*	289
**9**	*Hymenocrater bituminosus*	268
**10**	*Stachys byzantina*	1635
**11**	*Scutellaria tournefortii*	254
**12**	*Salvia macrosiphon*	233

*The herbarium specimens have been deposited at the Herbarium of Institute of Medicinal Plants, ACECR, Karaj, Iran.

## Methods

### Chemicals

All the chemicals used were purchased from Sigma-Aldrich Chemie Gmbh (Germany) and Merck (Germany) companies. The chemicals were of analytical grade. α-amylase activity was determined by measuring the absorbance of the mixtures at 540 nm in Elisa stat fax 2100 (Awarness Technology Inc).

### Plant materials

The plants were collected from different provinces in northern parts of Iran during June-July 2008–2011, and identified by Mr. Yousef Ajani. The herbarium specimens have been deposited at the Herbarium of Institute of Medicinal Plants, Jahade-Daneshgahi (ACECR), Karaj, Iran. The plant materials were cleaned and dried at shade and room temperature. All the scientific names and Herbarium voucher numbers of the plant materials used in this study are exhibited in Table 1. The plant materials were dried at shade in room temperature. Only the aerial parts of the plants were used for investigation.

### Extraction

Dried aerial parts of the plants were cut into small pieces and extracted with ethyl acetate, methanol and water–methanol 50:50 v/v at room temperature (24 h × 3 time) using percolation method. The extracts were filtered and concentrated by rotary evaporator, and ultimately dried by a freeze dryer.

### α-amylase inhibition

The α-amylase inhibition assay was performed by some modification in the method proposed by Giancarlo et al. [10]. The starch solution (1% w/v) was obtained by boiling and stirring 1 g of potato starch in 100 mL of sodium phosphate buffer for 30 min. The enzyme (EC 3.2.1.1; purchased from Sigma; soy bean source; It hydrolyses alpha bonds of large, alpha-linked polysaccharides, such as starch and glycogen, yielding glucose and maltose as the major form of amylase in humans and other mammals) solution (50 unit/1 mL) was prepared by mixing 0.01 g of α-amylase in 10 mL of sodium phosphate buffer (PH 6.9) containing 0.0006 mM sodium chloride. The extracts were dissolved in DMSO to give concentrations from 5 to 15 mg/mL (5, 10 and 15 mg/mL). The color reagent was a solution containing 0.1 g of 3,5-dinitrosalicylic acid plus 2.99 g sodium potassium tartrate in 0.16 g sodium hydroxide and phosphate buffer (10 mL).

Fifty microliter of each plant extract and 150 μL of starch solution as well as 10 μL of enzyme were mixed in a 96 well plate and incubated at 37°C for 30 min. Then, 20 μL of sodium hydroxide and 20 μL of colour reagent were added and the closed plate placed into a 100°C water bath. After 20 min, the reaction mixture was removed from the water bath and cooled, thereafter α-amylase activity was determined by measuring the absorbance of the mixture at 540 nm in Elisa stat fax 2100 (Awarness Technology Inc). Blank samples were used to correct the absorption of the mixture, in which the enzyme was replaced with buffer solution. Also, a control reaction was used, in which the plant extract was replaced with 50 μL of DMSO, and the maximum enzyme activity was determined. By removing the extract from solution, all the interferences from extract side will be removed such as colour or self-inhibitory interference with the assay. Acarbose solution at the concentrations (5, 10, 15 mg/mL) was used as a positive control. The inhibition percentage of α-amylase was assessed by the following formula:

$$ \mathrm{I}\alpha \hbox{--} \mathrm{Amylase}\ \%=100\times \left(\Delta {\mathrm{A}}_{\mathrm{control}} - \Delta {\mathrm{A}}_{\mathrm{sample}}\right)/\Delta {\mathrm{A}}_{\mathrm{control}} $$

$$ \Delta {\mathrm{A}}_{\mathrm{control}}={\mathrm{A}}_{\mathrm{test}}-{\mathrm{A}}_{\mathrm{Blank}} $$

$$ \Delta {\mathrm{A}}_{\mathrm{sample}}={\mathrm{A}}_{\mathrm{test}}-{\mathrm{A}}_{\mathrm{Blank}} $$

### Statistical analysis

Statistical analysis was performed using the SPSS version 21.0. The IC50 values were estimated by non-linear curve and presented as their respective 95% confidence limits. Probit analysis of variance was used to assess the presence of significant differences (p < 0.05) between the extracts.

## Results

In this study, sixteen extracts from 12 different species belonging to Lamiaceae family were evaluated for their possible α-amylase inhibitory activities alongside acarbose as a positive control. The α-amylase inhibitory activities and IC_50_ values of the acarbose and herbal extracs are summarized in Tables 2 and 3, respectively. As the results show, the inhibitory activities of all the extracts were varied from 1.9 to 18.6 (IC_50_, mg/mL). Moreover, the ethyl acetate extract of *P. bruguieri* (IC_50_ = 1.9 mg/mL) and the butanol extract of *P. persica* (IC_50_ = 3.6 mg/mL) exhibited the lowest IC_50_ values among all the species as the most potent herbal extracts. However, the inhibitory activity of *S. sahendica* (ethyl acetate extract), *S. macrosiphon* (ethyl acetate extract) and *P. caucasica* (butanol extract) on α-amylase was weak and their activities did not reach at least to the 50% of the enzyme inhibition level.

**Table 2:** **α-Amylase inhibitory activities and IC**_**50**_ **values of the acarbose**

**Compound**	**Concentration (mg/mL)**	**Inhibitory percentage ± SD**	**IC**_**50**_ **(mg/mL)**
Acarbose	5	69.9 ± 1.1	1.8
	10	96.1 ± 0.3	
	15	97.9 ± 0.8

**Table 3:** **α-Amylase inhibitory activities and IC**_**50**_ **values of the tested extracts**

**Plant species**	**Extracts**	**Concentration (mg/mL)**	**Inhibitory percentage ± SD**	**IC**_**50**_ **(mg/mL)**
***Phlomis kurdica***	Ethyl acetate	5	86.1 ± 7.3	16.2
		10	95.45 ± 3.3	
		15	84.5 ± 2.8	
***Phlomis bruguieri***	Ethyl acetate	5	58.5 ± 4.8	1.9
		10	67.6 ± 7.5	
		15	80.1 ± 6.4	
	Methanol	5	59.6 ± 1.9	9.2
		10	38.2 ± 3.7	
		15	49.7 ± 5.7	
***Phlomis rigida***	Ethyl acetate	5	40 ± 5.2	7.8
		10	57.8 ± 6.4	
		15	73.5 ± 1.6	
	Methanol	5	58.2 ± 1.8	4.4
		10	66.6 ± 0.1	
		15	94.8 ± 3.3	
	Methanol–water	5	56.5 ± 4.8	5.7
		10	48.3 ± 2.9	
		15	89 ± 4.7	
***Phlomis olivieri***	Butanol	5	20.7 ± 0.4	12.8
		10	31.3 ± 1.8	
		15	63 ± 3.6	
***Phlomis anisodonta***	Butanol	5	27.9 ± 2.3	18.6
		10	31.9 ± 2.6	
		15	44.8 ± 3.4	
***Phlomis persica***	Butanol	5	59.3 ± 1.6	3.6
		10	85.8 ± 1.2	
		15	97.2 ± 1.6	
***Phlomis caucasica***	Butanol	5	75.7 ± 2.9	_
		10	92.6 ± 0.6	
		15	92.4 ± 1.1	
***Satureja sahendica***	Ethyl acetate	5	83.8 ± 2.9	_
		10	80.9 ± 0.8	
		15	83.6 ± 1	
	Methanol–water	5	16.9 ± 2	8.5
		10	73.7 ± 1.4	
		15	88.3 ± 3.8	
***Salvia macrosiphon***	Ethyl acetate	5	57.1 ± 2.5	_
		10	66.5 ± 2.5	
		15	67.9 ± 2.7	
***Hymenocrater bituminosus***	Ethyl acetate	5	22.5 ± 4.5	6.6
		10	97.7 ± 0.3	
		15	97 ± 1.7	
***Stachys byzantina***	Methanol	5	39.2 ± 2.7	5.8
		10	84.1 ± 2.5	
		15	86.1 ± 2.8	
***Scutellaria tournefortii***	Methanol	5	36.9 ± 0.1	13.5
		10	45 ± 0.1	
		15	52.1 ± 0.1	

A concentration dependent inhibition was observed for various concentrations of each herbal extract except for *P. kurdica* and *S. sahendica* (ethyl acetate extracts). For these herbal extracts, the highest inhibitory activities were found to be 95.4 ± 3.3 (at 10 mg/mL) and 83.8 ± 2.9 (at 5 mg/mL), respectively (Table 3). The ethyl acetate extract of *H. bituminosus* (97.7 ± 0.3) at a concentration of 10 mg/mL and the butanol extract of *P. persica* (97.2 ± 1.6) at a concentration of 15 mg/mL showed the greatest inhibitory activities. The literature reveals that this phenomenon is probably due to conformational change from binding of compounds to the enzyme by increased concentration [11,12].

## Discussion

Among the plants used, *Satureja* genus, called “Savory” in English and “Marzeh” in Persian, consists of about 14 species in Iran as the herbaceous perennial herbs, nine species of which are endemic in Iran [13]. The species of this genus are well-known for antioxidant, anti-inflammatory, cholinesterase inhibitory activities, anti-fungal, anti-microbial, anti-spasmodic and anti-diarrhoea effects [14,15], and also in the past time they were used in Europe as an anti-thirst in diabetes [16]. *S. Sahendica* is one of the endemic species, which was already subjected to phytochemical investigation on both extract and essential and predicted to exhibit anti-diabetic activity due to the presence of *beta-sitosterol* [14,16,17]. Our previous investigation on this species demonstrated the presence of luteolin together with oleanolic acid, beta-sitosterol and diosmetin, which were isolated from the ethyl acetate and methanol extracts of *S. sahendica* [14]*.* Additionally, the hydro-distilled oil of *S. sahendica* was rich of monoterpenes with thymol (37.2%), p-cymene (32.6%) and γ-terpinene (11.5%) as the major compounds. Although we expected a higher inhibitory activity for this plant due to the presence of beta-sitosterol and oleanolic acid [17], however, the results revealed that it may not effect via α-amylase inhibitory activity, and other mechanisms should be considered for further studies on this species. Also, it might be due to the enzyme source, soy bean, and the inhibitory activity may evaluate differently by using other sources of α-amylase like pancreatic enzyme.

Another genus *Salvia* has 58 species in Iran and composes of bioactive compounds such as flavonoids and polyphenols with characteristic cholinesterase-inhibitory, anti-inflammatory and antioxidant activities. *S. macrosiphon* is one this genus investigated for its phytochemical contents and demonstrated to be rich of beta-sitosterol and flavones as well as flavone glycosides [18]. This species was also not effective to inhibit α-amylase enzyme. Furthermore, *Hymenocrater* genus consists of 11 species that nine of them are growing in Iran and surprisingly possess antioxidant properties as well as anti-diabetic, anti-clotting, anti-inflammatory and anti-cancer activities, because of the presence of two important metabolites: rosmarinic acid and rutin in the areal branches [19,20]. Although *H. bituminosus* shows the highest α-amylase inhibitory activity in this study, its phytochemistry is not exactly determined and more investigations by the guide of α-amylase inhibitory activity are recommended.

Eventually, *Phlomis* genus is native to East Mediterranean from Central Asia to China. Bibliography revealed that the methanolic extract of the aerial parts of *P. anisodonta* exhibited lipid peroxidation, antioxidant and anti-diabetic activities [21]. Furthermore, the methanolic extract of the aerial parts of *P. persica* caused a dramatic decrease in fasting blood glucose while the insulin levels augmented. Also, the prevention of diabetes due to losing weight is reported from this herbal medicine [22].

Regarding the importance of *Phlomis* species as the candidates for future anti-diabetic medicines, a bibliography on its phytochemistry revealed that different species of this genus are rich in iridoids, flavonoids, terpenoids, phenolic compounds and their glycosides, which are supposed to be related to various biological and pharmacological effects of them including anti-nociceptive, antioxidant, antimicrobial and anti-diabetic effects [23]. Moreover, methanolic extract of the aerial parts of *P. olivieri* has been reported to include chrysoeriol-7-O-β-D-glucoside and verbascoside. Additionally, two flavonoid glycosides, chrysoeriol-7-β-D- (3''-E-p-coumaroyl) glucoside and one iridoid glycoside, lamiide, were already isolated and identified from a methanolic extract of the aerial parts of *P. persica* [24]*.* To the best of our knowledge, none of the identified compounds in these species have been studied for α-amylase inhibitory activity so far. Furthermore, there is no report on chemical constituents of *P. bruguieri* from Iran except on its essential oil that revealed to compose of germacrene D (23.6%), 4-hydroxy-4-methyl-2-pentanone (15.0%), α-pinene (6.8%) and β-caryophyllene (6.7%) [25].

Taking together, the present study demonstrated that just some of the *Phlomis* species showed a strong α-amylase inhibitory activity including *P. bruguieri* and *P. persica.* Nevertheless *P. anisodonta* was already reported for its anti-diabetic activity, was not able to inhibit α-amylase activity sufficiently. However, the present study has been carried out on a "model alpha-amylase" and the results should be checked in future studies on mammalian amylase or *in vivo* models.

## Conclusions

In conclusion, among all the herbal extracts tested in this study, *P. bruguieri* and *P. persica* can be the promising sources of α-amylase inhibitors. However, *P. rigida, S. bizantina* and *H. bituminosus* that exhibited moderate activity can be stand on second level of interest.

## References

[1] Abesundara KJ, Matsui T, Matsumoto K (2004). Alpha-glucosidase inhibitory activity of some Sri Lanka plant extracts, one of which, *Cassia auriculata*, exerts a strong antihyperglycemic effect in rats comparable to the therapeutic drug acarbose. J Agric Food chem.

[2] **[WHO] World Health Organization: 10 facts about diabetes.** 2011 [www.who.int/diabetes/en/.accessed]

[3] Mathers CD, Loncar D: **Projections of global mortality and burden of disease from 2002 to 2030.** *PLoS Med* 2006, **3:**e442. 10.1371/journal.pmed.0030442 PMC1664601 17132052

[4] WHO World Health Organization (2011). Global status report on noncommunicable diseases 2010.

[5] [WHO] World Health Organization: How can the burden of diabetes be reduced. 2014. **[**http://www.who.int/mediacentre/factsheets/fs312/en/index.html]

[6] Jarald E, Joshi SB, Jain DC (2008). Diabetes and herbal medicines. Int J Pharm Tech.

[7] Ghadyale V, Takalikar S, Haldavnekar V, Arvindekar A: **Effective control of post prandial glucose level through inhibition of intestinal alpha glucosidase by*****Cymbopogon martini*****(Roxb).** *Evid Based Complement Alternat Med* 2011, **2012:**372909. 10.1155/2012/372909 PMC3139892 21792369

[8] Geethalaksrihmi R, Sarada DVL, Marimuthu P, Ramasamy K (2010). α-Amylase inhibitory activity of *Trianthema decandral*. Int J Biotech Biochem.

[9] Youn JY, Park HY, Cho KH (2004). Anti-hyperglycemic activity of *Commelina communis* L.: inhibition of alpha-glucosidase. Diabetes Res Clin Pract.

[10] Giancarlo S, Rosa LM, Nadjafi F, Francesco M (2006). Hypoglycaemic activity of two spices extracts: *Rhus coriaria* L. and *Bunium persicum* Boiss. Nat Prod Res.

[11] Andrade-Cetto A, Becerra-Jimenez J, Cardenas-Vazquez R (2008). Alpha-glucosidase inhibiting activity of some Mexican plants used in the treatment of type 2 diabetes. J Ethnopharmacol.

[12] Kim JS, Kwon CS, Son KH (2000). Inhibition of alpha-glucosidase and amylase by luteolin, a flavonoid. Biosci Biotechnol Biochem.

[13] Rechinger KH (1982). Flora Iranica.

[14] Saeidnia S, Nourbakhsh MS, Gohari AR, Davood A (2011). Isolation and identification of the main compounds of *Satureja sahendica* Bornm. Aust J Basic Appl Sci.

[15] Balali P, Soodi M, Saeidnia S (2012). Protective effect of some medicinal plants from Lamiaceae family against Beta amyloid induced toxicity in PC12 cell. Tehran Univ Med J.

[16] Gohari AR, Nourbakhsh MS, Saeidnia S (2011). Comparative investigation of the volatile oils of *Satureja sahendica* Bornm., extracted via steam and hydro distillations. J Essent Oil Bear Plants.

[17] Saeidnia S, Manayi A, Gohari AR, Abdollahi M (2014). The story of beta-sitosterol-a review. Eur J Med Plants.

[18] Gohari AR, Ebrahimi H, Saeidnia S, Foruzani M, Ebrahimi P, Ajani Y (2011). Flavones and flavone glycosides from *Salvia macrosiphon* Boiss. Iran J Pharm Res.

[19] Gohari AR, Saeidnia S, Shahverdi AR, Yassa N, Malmir M, Mollazade K, Naghinejad AR (2009). Phytochemistry and antimicrobial compounds of *Hymenocrater calycinus*. Eur Asia J Bio Sci.

[20] Gohari AR, Saeidnia S, Hajimehdipoor H, Shekarchi M, Hadjiakhoondi A (2011). Isolation and quantification of rosmarinic acid from *Hymenocrater calycinus*. J Herbs Spices Med Plants.

[21] Sarkhail P, Rahmanipour S, Fadyevatan S, Mohammadirad A, Dehghan G, Amin G, Shafiee A, Abdollahi (2007). Antidiabetic effect of *Phlomis anisodonta*: effects on hepatic cells lipid peroxidation and antioxidant enzymes in experimental diabetes. Pharmacol Res.

[22] Sarkhail P, Abdollahi M, Fadayevatan S, Shafiee A, Mohammadirad A, Dehghan G, Esmaily H, Amin G (2010). Effect of *Phlomis persica* on glucose levels and hepatic enzymatic antioxidants in streptozotocin-induced diabetic rats. Pharmacogn Mag.

[23] Sarkhail P, Nikan M, Sarkheil P, Gohari AR, Ajani Y, Hosseini R, Hadjiakhoondi A, Saeidnia S: **Quantification of verbascoside in medicinal species of Phlomis and their genetic relationships.** *Daru* 2014, **22:**32. 10.1186/2008-2231-22-32 PMC3998186 24650578

[24] Sarkhail P, Monsef-Esfehani H, Amin G, Salehi-Surmaghi MH, Shafiee A (2006). Phytochemical study of Phlomis olivieri benth. and Phlomis persica boiss. Daru.

[25] Morteza-Semnani K, Saeedi M (2005). The essential oil composition of *Phlomis bruguieri* Desf. from Iran. Flav Frag J.

